# German surgeons’ perspective on the application of artificial intelligence in clinical decision-making

**DOI:** 10.1007/s11548-025-03326-z

**Published:** 2025-02-05

**Authors:** Jonas Henn, Tijs Vandemeulebroucke, Simon Hatterscheidt, Jonas Dohmen, Jörg C. Kalff, Aimee van Wynsberghe, Hanno Matthaei

**Affiliations:** 1 Department of General, Visceral, Thoracic and Vascular Surgery, University Hospital Bonn, Venusberg-Campus 1, 53127 Bonn, Germany; 2 Bonn Surgical Technology Center (BOSTER), University Hospital Bonn, Bonn, Germany; 3 Bonn Sustainable AI Lab, Institute of Science and Ethics, University of Bonn, Bonn, Germany

**Keywords:** Artificial intelligence, Machine learning, Clinical decision-making, Surgery, Acute abdominal pain

## Abstract

**Purpose:**

Artificial intelligence (AI) is transforming clinical decision-making (CDM). This application of AI should be a conscious choice to avoid technological determinism. The surgeons’ perspective is needed to guide further implementation.

**Methods:**

We conducted an online survey among German surgeons, focusing on digitalization and AI in CDM, specifically for acute abdominal pain (AAP). The survey included Likert items and scales.

**Results:**

We analyzed 263 responses. Seventy-one percentage of participants were male, with a median age of 49 years (IQR 41–57). Seventy-three percentage of participants carried out a senior role, with a median of 22 years of work experience (IQR 13–28). AI in CDM was seen as helpful for workload management (48%) but not for preventing unnecessary treatments (32%). Safety (95%), evidence (94%), and usability (96%) were prioritized over costs (43%) for the implementation. Concerns included the loss of practical CDM skills (81%) and ethical issues like transparency (52%), patient trust (45%), and physician integrity (44%). Traditional CDM for AAP was seen as experience-based (93%) and not standardized (31%), whereas AI was perceived to assist with urgency triage (60%) and resource management (59%). On median, generation Y showed more confidence in AI for CDM (*P* = 0.001), while participants working in primary care hospitals were less confident (*P* = 0.021).

**Conclusion:**

Participants saw the potential of AI for organizational tasks but are hesitant about its use in CDM. Concerns about trust and performance need to be addressed through education and critical evaluation. In the future, AI might provide sufficient decision support but will not replace the human component.

**Supplementary Information:**

The online version contains supplementary material available at 10.1007/s11548-025-03326-z.

## Introduction

### Artificial intelligence-driven decision support in surgery

Clinical reasoning or clinical decision-making (CDM) is a crucial task in the daily work of every physician [1]. Surgeons regularly face complex CDM under adverse conditions, such as assessing the need for urgent surgery in patients with acute abdominal pain (AAP) [2]. Therefore, clinical research has explored clinical decision support systems (CDSS) to improve surgical CDM. However, conventional systems are hindered by manual data entry and limited accuracy [3]. In contrast, artificial intelligence (AI) is increasingly recognized for its potential to revolutionize surgical CDM by facilitating data-driven decisions [3]. A review of the use of machine learning (ML) indicated its potential superiority over conventional CDM in abdominal surgery [4]. *Brennan *et al*.*, for example, demonstrated the potential of ML in surgical risk assessment. Their ML algorithm outperformed surgeons in predicting postoperative morbidity and mortality. Moreover, it has been shown that interaction with an algorithm improved physicians’ clinical assessment [5].

### Challenges in the application of AI in surgical CDM

Despite these advances, AI-driven CDSS are still in early stages of adoption due to challenges such as data standardization, interpretability, safety, and ethical concerns [3, 4, 6]. From an ethical point of view, the application of AI should be a conscious and informed choice, not an inevitable outcome pushed from outside the healthcare domain. To avoid this technological determinism, the integration of AI in surgical CDM necessitates thoughtful consideration and an inclusive dialog among all stakeholders (i.e., physicians, patients, data scientists, etc.) regarding its ethical implications and optimal implementation [6]. A recent survey indicated that half of the questioned emergency surgeons are interested in AI applications within their field [7]. These perspectives of primary users are crucial for guiding the further development of AI-driven CDSS in surgery. Existing literature, however, offers limited insight. For example, the ARIES project explored general perceptions of AI’s potential in combination with robotics [7], and *Cobianchi *et al*.* focused on the perception of conventional CDM and only assessed the application of AI in a limited way [8]. Crucially, neither study addressed ethical concerns nor incorporated interdisciplinary perspectives. Moreover, how AI will be implemented and the success of it will be impacted by national and regional healthcare systems [6]. To our knowledge, German perspectives on the usefulness, implementation, and ethics of AI in CDM are nonexistent. Hence, we set up a survey to create insight into German surgeons’ perspectives on the use of AI in CDM. The survey aimed to identify obstacles, ethical concerns, and areas needing improvement before AI can be widely adopted in clinical practice.

## Materials and methods

### Design and setting

We followed the Checklist for Reporting Results of Internet E-Surveys (CHERRIES) and implemented its recommendations as applicable (Online Resource 1) [9]. We conducted an open online survey, and invited members of the German Society for General and Visceral Surgery (DGAV). The invitation email provided details on the survey’s objectives, background, and an estimated time frame of approximately five minutes. Participation was voluntary, and no incentives were offered. We internally tested the survey for usability and technical functionality. The online questionnaire was created with SoSci Survey version 3.4.22 (SoSci Survey GmbH, Munich, Germany) and was accessible at www.soscisurvey.de from November 9th to December 15th, 2023. To protect data privacy, we chose not to use technical measures such as cookies, IP addresses, or fingerprinting to prevent multiple participations. No further promotion of the survey was conducted beyond the initial invitation. Participants remained anonymous and provided informed consent before starting the survey.

### Survey

The survey and its items were developed through an open discussion within an interdisciplinary research group of surgeons and ethicists. The survey was made available in German. The complete questionnaire and its English translation are available in Online Resource 2. In total, 58 unique items were presented across three parts and six screens. Firstly, we collected demographic information, asking participants for their gender, age, and years of experience as a physician. Participants also specified the level of the hospital they work at: primary, secondary, tertiary, based on the German *Versorgungsstufen* (Online Resource 2), and their position within the hospital: junior (resident and attending) or senior (consultant and chief/director). Secondly, we asked participants about their perspectives on the current and future state of digitalization in healthcare. We also asked participants about their views on potential opportunities and concerns regarding the implementation of AI in CDM. Finally, we explored the participants’ viewpoints on surgical CDM for patients with AAP and the potential role of AI in CDM for these patients. No consistency or completeness checks were performed before the submission. Participants could review their answers and go back within the questionnaire.

### Technical analysis

In addition to participants’ characteristics, we used Likert items throughout the survey. For the questions “Which chances do you see for the application of AI in CDM?”, “Regarding AI in CDM, do you share concerns regarding …?”, and “For patients with AAP, AI could …?”, we combined responses into composite Likert scales and calculated each participant’s mean score for group comparison. We grouped participants by demographic characteristics, with widely accepted definitions of the generations [10]. Differences between groups were analyzed using the Kruskal–Wallis test for comparisons involving more than two groups and the Mann–Whitney U test for pairwise comparisons. Descriptive and inferential statistics were calculated using R software (R Foundation for Statistical Computing, Vienna, Austria) and RStudio version 2023.12.0 Build 369 (Posit Software, PBC, Boston, USA). The generative AI tools DeepL (DeepL SE, Cologne, Germany) and ChatGPT (OpenAI, San Francisco, USA, RRID:SCR_023775) were used to prepare the manuscript: minor parts of the manuscript were translated from German to English, and the AI generated a first draft of the abstract.

## Results

### Participants

Of the 294 participants who began the survey, 263 reached the final page, yielding a participation rate of 88%. The median completion time was 329 s (IQR 260–460), and the median completeness rate was 100% (IQR 98.3–100). All questionnaires where the last page was accessed were included, regardless of completeness. Table 1 summarizes the characteristics of the participants: most individuals (71%) were male, with a median age of 49 years (IQR 41–57). A majority held senior positions (73%), with a median of 22 years of experience (IQR 13–28). The distribution of hospital levels was relatively balanced (Primary: 37%; Secondary: 30%; Tertiary: 29%).

**Table 1:** Characteristics of participants

All (N, %)	263	100
Age (Median, IQR)	49	41–57
Experience (Median, IQR)	22	13–28
Sex (N, %)		
Female	73	28
Male	187	71
NA	3	1
Generation (N, %)		
Boomer (1946—1964)	54	21
X (1965—1979)	123	47
Y (1980—1994)	85	32
NA	1	0
Position (N, %)		
Junior	60	23
Senior	193	73
NA	10	4
Hospital (N, %)		
Tertiary	75	29
Secondary	79	30
Primary	98	37
NA	11	4

Continuous variables are displayed with median and interquartile range (IQR). Categorical variables are displayed with absolute (N) and relative (%) frequency

NA: Not available

### Digitalization in healthcare

**Fig. 1:**
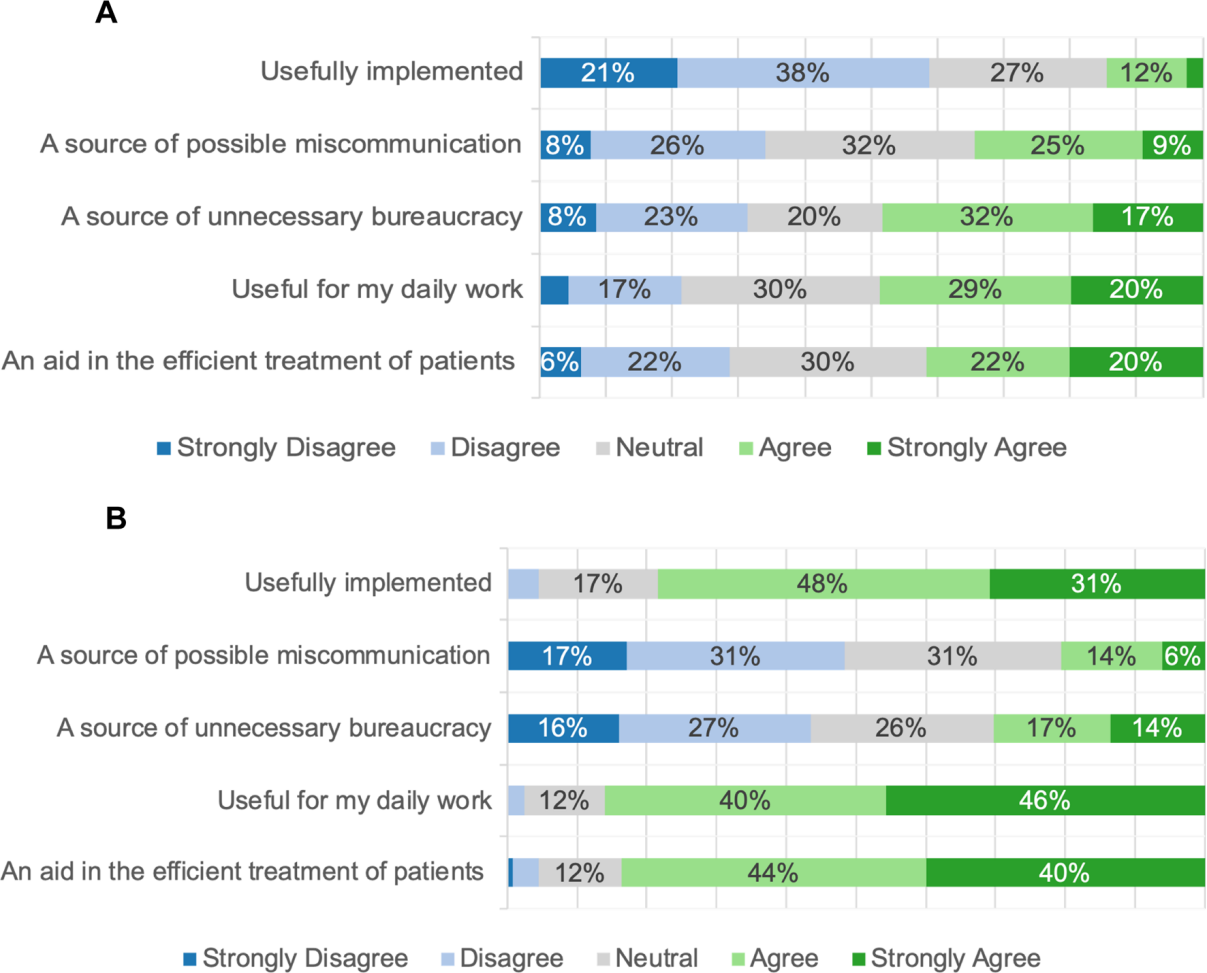
**A** Stacked bar plot showing the responses to the general question: “Digitalization in Healthcare right now is…?”. The sub-questions are displayed on the y-axis, and the responses are displayed on the x-axis. The size of the bars represents the frequency of the response in %. For readability, values below 6% are not shown. **B** Stacked bar plot showing the responses to the general question: “Digitalization in Healthcare in the future will be…?”. The sub-questions are displayed on the y-axis, and the responses are displayed on the x-axis. The size of the bars represents the frequency of the response in %. For readability, values below 6% are not shown

Participants perceived digitalization as beneficial for their daily work (49%) but expressed concerns regarding useful implementation (59%) and the potential for increased bureaucracy (49%). Nonetheless, they were optimistic about future improvements in digitalization (79%) and less apprehensive about associated bureaucratic impacts (31%). For details, see Fig. 1A and B.

### AI in CDM

**Fig. 2:**
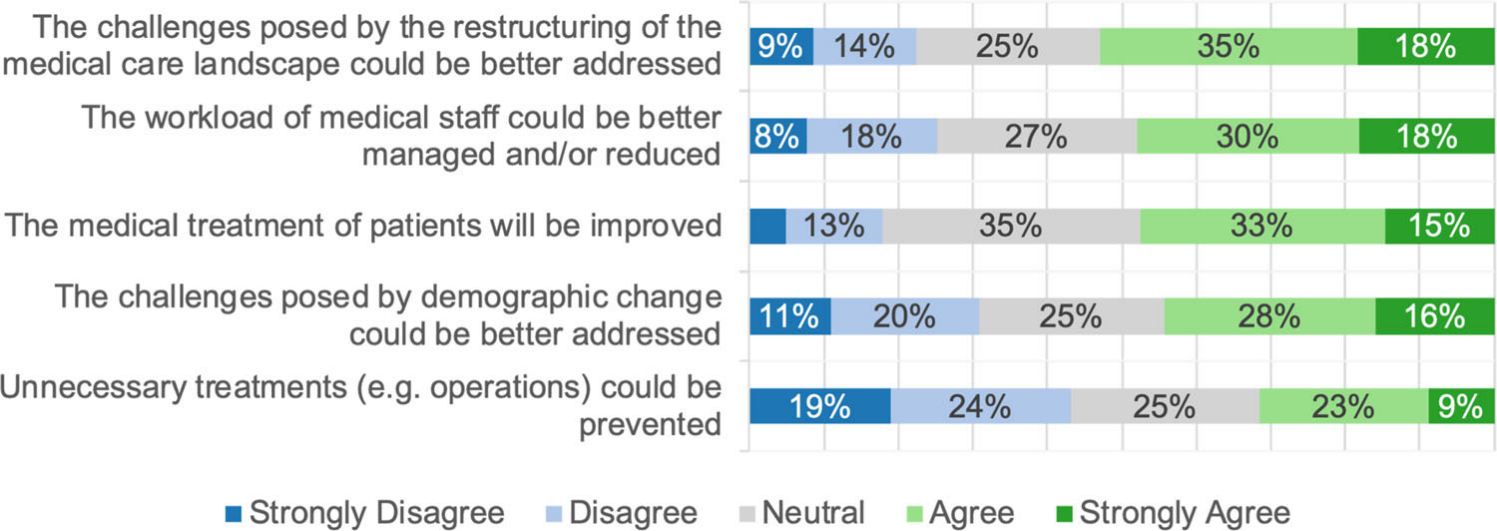
Stacked bar plot showing the responses to the general question: “Which chances do you see for the application of AI in CDM?”. The sub-questions are displayed on the y-axis, and the responses are displayed on the x-axis. The size of the bars represents the frequency of the response in %. For readability, values below 6% are not shown

**Fig. 3:**
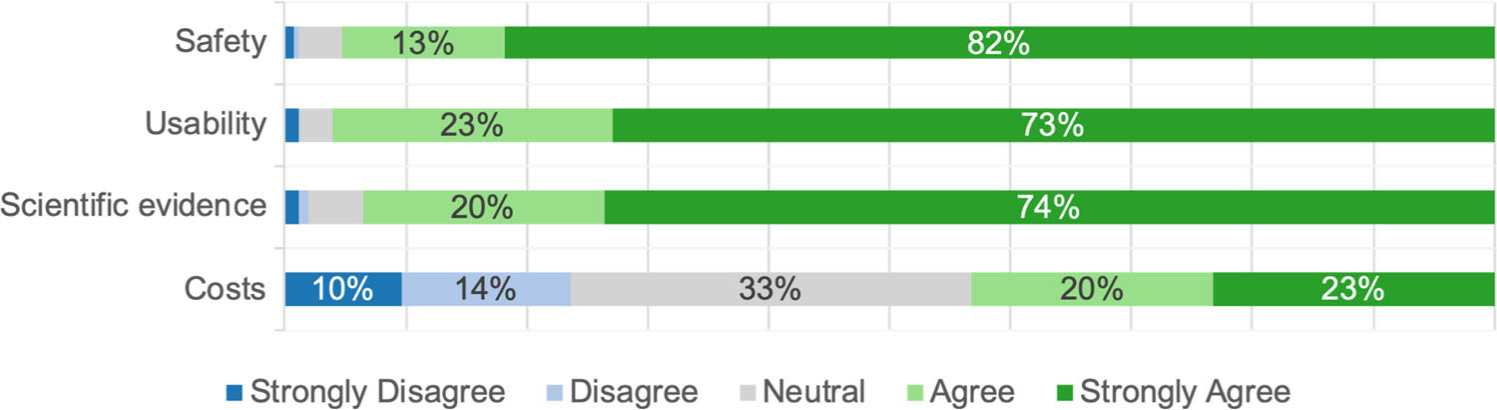
Stacked bar plot showing the responses to the general question: “Which parameters are important to you for the implementation of AI in CDM?”. The sub-questions are displayed on the y-axis, and the responses are displayed on the x-axis. The size of the bars represents the frequency of the response in %. For readability, values below 6% are not shown

Participants rated AI’s potential in CDM as moderate to good for supporting organizational tasks and managing workload (48–53%; see first three questions) but were less optimistic about its ability to prevent unnecessary treatments (31%) (Fig. 2). If AI was to be implemented in CDM, participants equally prioritized safety (95%), usability (96%), and scientific evidence (94%) as key considerations. In comparison, the participants much less addressed concerns regarding possible costs (43%) (Fig. 3).

### Concerns regarding AI in CDM

**Fig. 4:**
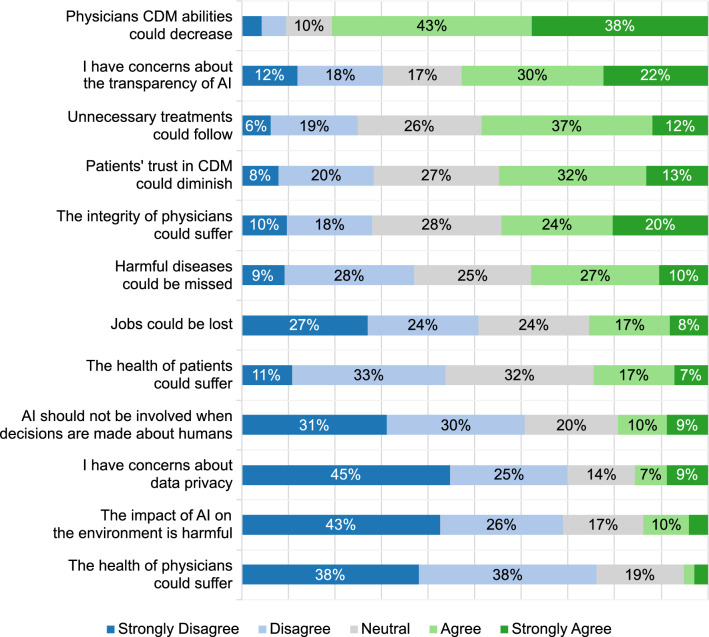
Stacked bar plot showing the responses to the general question: “Regarding AI in CDM, do you share concerns regarding …?”. The sub-questions are displayed on the y-axis, and the responses are displayed on the x-axis. The size of the bars represents the frequency of the response in %. For readability, values below 6% are not shown

Participants agreed with several concerns when applying AI for CDM, with the primary fear being a loss of their own CDM abilities (81%). Additional concerns included the risk of unnecessary treatments (49%) and ethical issues regarding AI’s transparency (53%), patient trust in CDM (45%), and physician integrity (44%). In contrast, concerns related to personal health (5%) and job security (25%) were not prominent. Furthermore, most participants did not view data privacy (16%) or the environmental impact of AI systems (14%) as significant issues (Fig. 4).

### AI in CDM for AAP

**Fig. 5:**
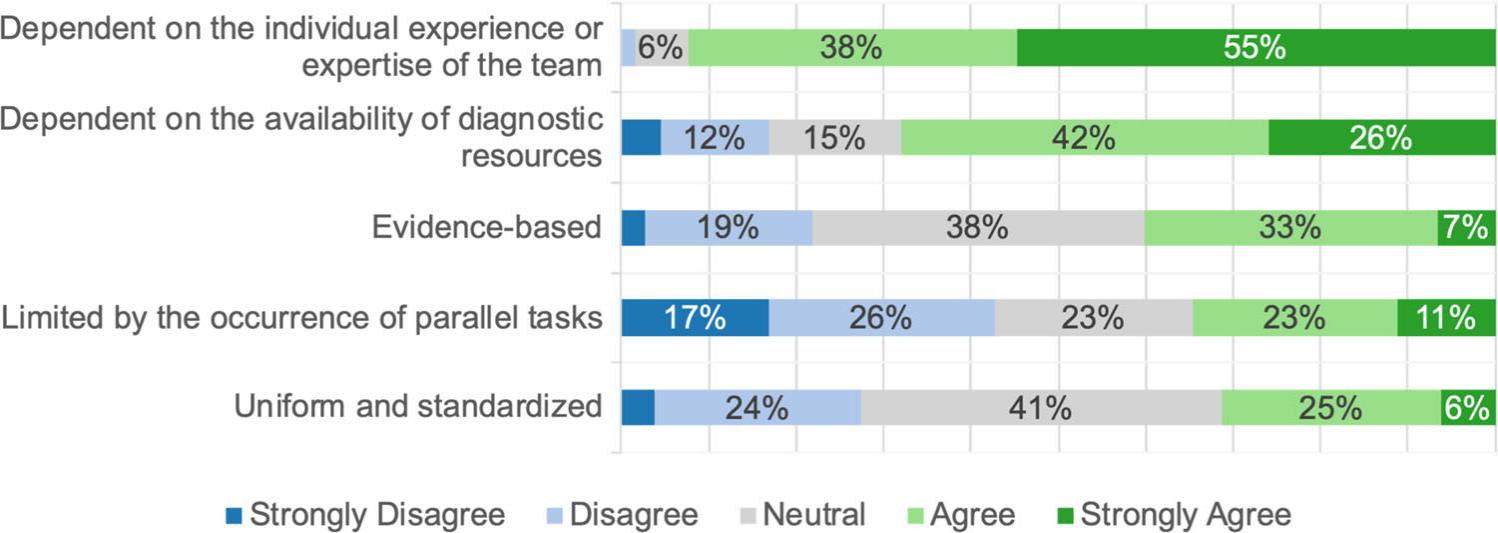
Stacked bar plot showing the responses to the general question: “How do you experience clinical decision-making for acute abdominal pain? The process is …?”. The sub-questions are displayed on the y-axis, and the responses are displayed on the x-axis. The size of the bars represents the frequency of the response in %. For readability, values below 6% are not shown

**Fig. 6:**
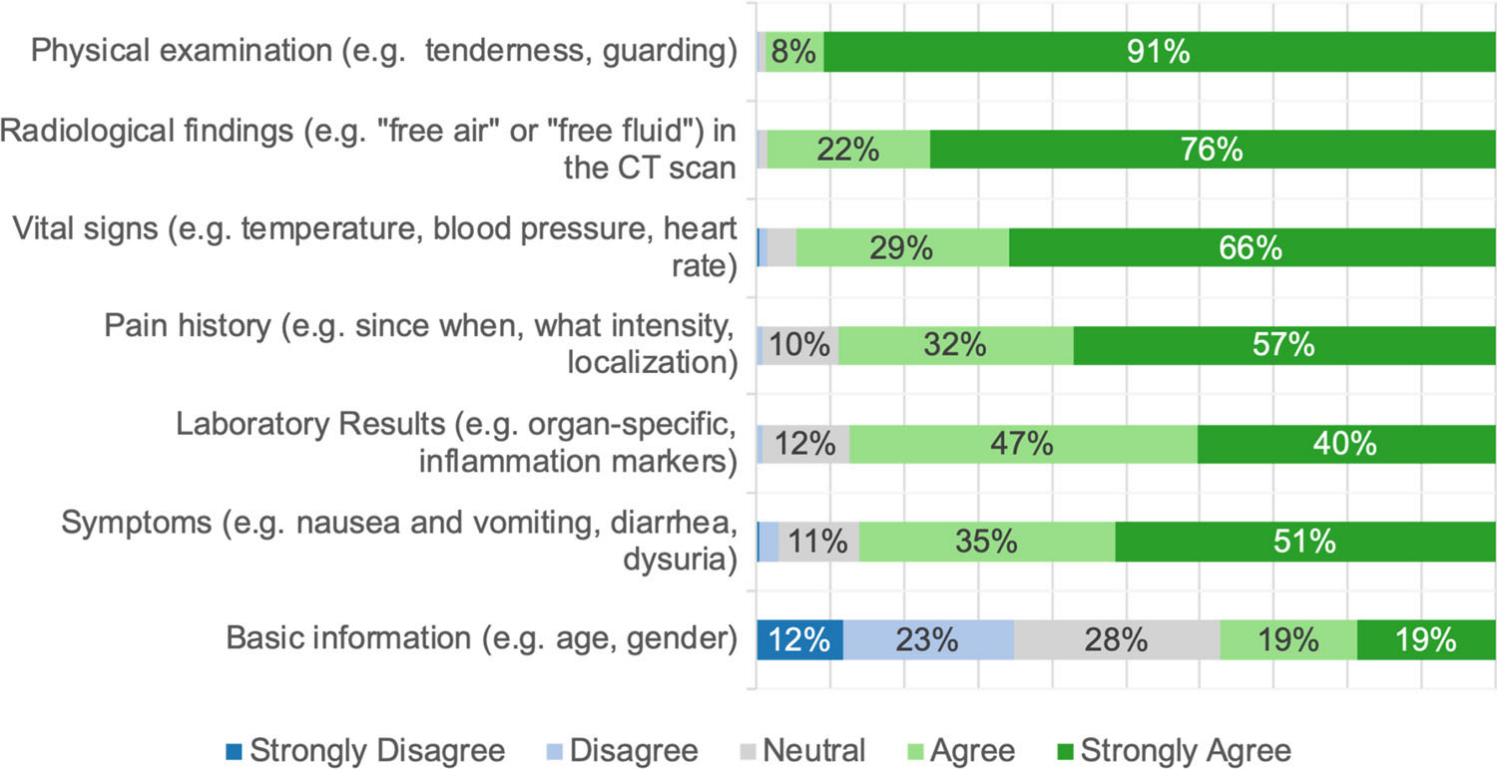
Stacked bar plot showing the responses to the general question: “How important do you consider the following variables in the assessment of whether an urgent (i.e., within 24 h) operation is necessary?”. The sub-questions are displayed on the y-axis, and the responses are displayed on the x-axis. The size of the bars represents the frequency of the response in %. For readability, values below 6% are not shown

Participants perceived traditional CDM for AAP as insufficiently standardized (31%) and not evidence-based (40%), resulting in heavy reliance on individual experience (92%) (Fig. 5). Participants rated physical examination (99%) as the most important factor in CDM for AAP, closely followed by radiological findings (98%) (Fig. 6).

**Fig. 7:**
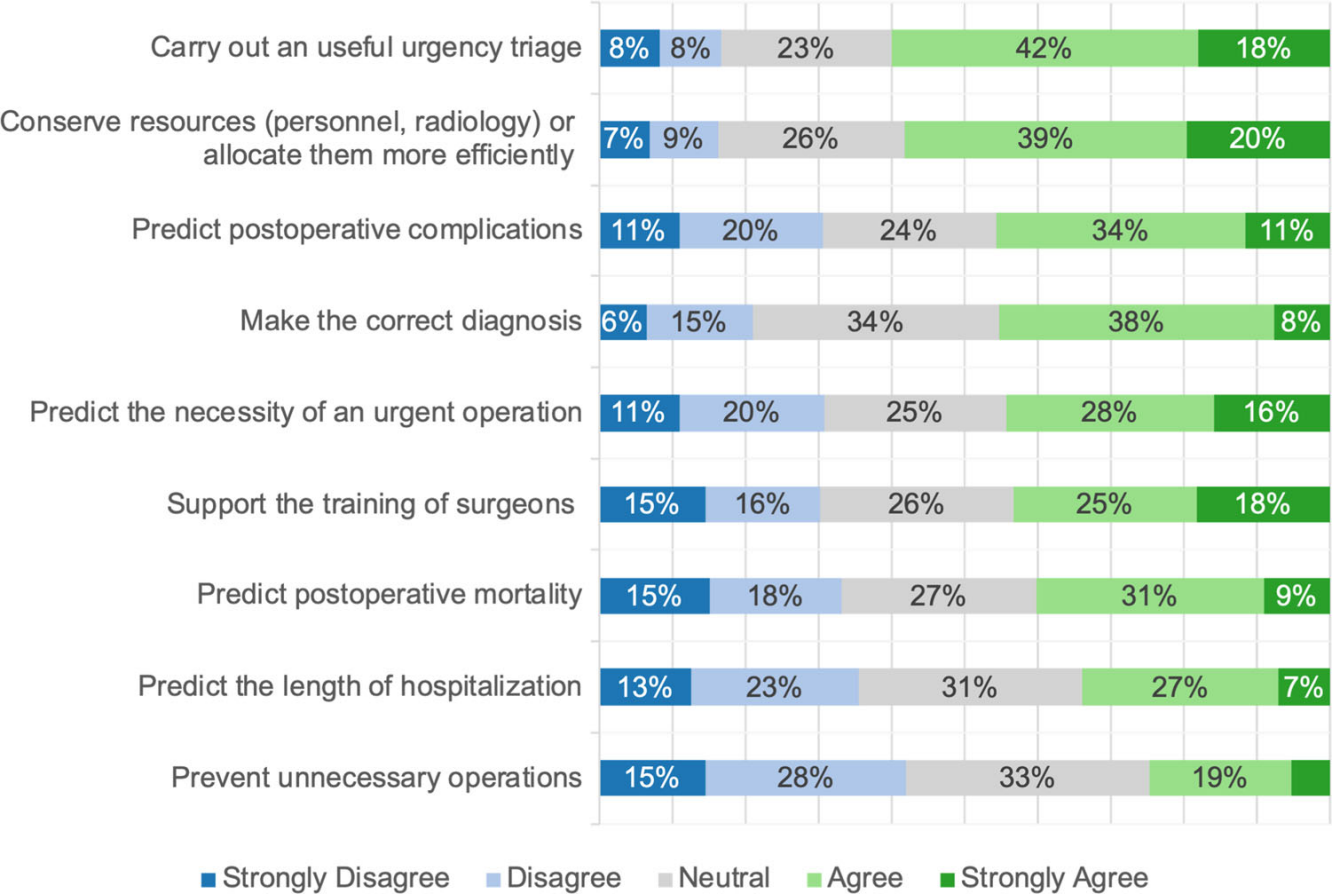
Stacked bar plot showing the responses to the general question: “For patients with AAP, AI could …?”. The sub-questions are displayed on the y-axis, and the responses are displayed on the x-axis. The size of the bars represents the frequency of the response in %. For readability, values below 6% are not shown

The participants believed that AI could help conserve and manage resources (58%) and assist with urgency triage for patients with AAP (60%). However, they expressed lower confidence in AI’s ability to make the right diagnoses (45%) or to predict medical events such as complications (46%), mortality (40%), and hospitalization (34%). Like perceptions of AI in clinical decision-making (CDM) in general, participants were most skeptical about AI’s potential to prevent unnecessary treatments (25%) (Fig. 7).

### Intergroup differences

Table 2 displays the median values for each Likert scale, stratified by group. Generation Y demonstrated higher median confidence in AI’s potential for CDM in general (Boomer 3.2, Generation X 3.0, Generation Y 3.6, *P* = 0.001) and for AAP (Boomer 3.2, Generation X 3.1, Generation Y 3.4, *P* = 0.006). Participants working in primary care hospitals showed lower median confidence in AI’s potential for CDM in general (Primary 3.0, Secondary 3.4, Tertiary 3.5, *P* = 0.021) and for AAP (Primary 3.0, Secondary 3.2, Tertiary 3.5, *P* = 0.012). We observed no relevant differences in the median values for concerns, except those participants working in primary care expressed more concerns than those in tertiary hospitals (Primary 3.1, Tertiary 2.8, *P* = 0.035, data for pairwise comparison not shown in Table 2). Finally, we observed a difference of the median between sexes regarding the perceived possibilities of AI for AAP (female 3.1, male 3.3, *P* = 0.030).

**Table 2:** Comparison between groups for the Likert scales “Chances of AI in CDM” (Fig. 2), “Possibilities of AI for AAP” (Fig. 7), and “Concerns regarding AI in CDM” (Fig. 4). Table shows the median value (interquartile range)

Generation	All	Boomer	X	Y	*P*
Chances of AI in CDM	3.2 (2.6–3.9)	3.2 (2.6–3.8)	3.0 (2.4–3.6)	3.6 (3.0–4.0)	0.001
Possibilities of AI for AAP	3.2 (2.7–3.7)	3.2 (2.9–3.7)	3.1 (2.5–3.6)	3.4 (2.9–4.0)	0.006
Concerns regarding AI in CDM	3.0 (2.4–3.4)	3.0 (2.3–3.6)	3.1 (2.4–3.5)	2.9 (2.4–3.2)	0.359

Type of hospital	All	Primary	Secondary	Tertiary	*P*
Chances of AI in CDM	3.2 (2.6–3.9)	3.0 (2.2–3.8)	3.4 (2.8–3.8)	3.5 (2.7–4.0)	0.021
Possibilities of AI for AAP	3.2 (2.7–3.7)	3.0 (2.4–3.6)	3.2 (2.8–3.7)	3.4 (2.9–3.9)	0.012
Concerns regarding AI in CDM	3.0 (2.4–3.4)	3.1 (2.4–3.5)	3.0 (2.3–3.3)	2.8 (2.3–3.2)	0.089

Sex	All	Female	Male	*P*
Chances of AI in CDM	3.2 (2.6–3.9)	3.2 (2.4–3.8)	3.4 (2.6–4.0)	0.578
Possibilities of AI for AAP	3.2 (2.7–3.7)	3.1 (2.6–3.4)	3.3 (2.8–3.8)	0.030
Concerns regarding AI in CDM	3.0 (2.4–3.4)	3.0 (2.5–3.5)	2.9 (2.4–3.4)	0.236

Position	All	Junior	Senior	*P*
Chances of AI in CDM	3.2 (2.6–3.9)	3.5 (2.8–4.0)	3.2 (2.6–3.8)	0.125
Possibilities of AI for AAP	3.2 (2.7–3.7)	3.3 (2.9–3.8)	3.2 (2.7–3.7)	0.456
Concerns regarding AI in CDM	3.0 (2.4–3.4)	3.0 (2.5–3.3)	2.9 (2.3–3.4)	0.477

## Discussion

Our survey provides detailed insights into German surgeons’ perspectives on the use of AI in CDM in general and for patients with AAP. This must be compared with current literature to derive recommendations for fruitful action.

### AI for CDM: participants’ perspectives and scientific evidence

Our participants perceived the greatest potential for AI in CDM to support organizational tasks such as urgency triage, resource management, or handling workload (Figs. 2 and 7). A representative survey conducted by Deloitte GmbH revealed that 47% of the German population expect the use of AI in medicine will result in increased time for doctor-patient interactions by optimizing medical processes [11]. Both patients and our participants appear to agree with current scientific approaches in this matter. For instance, *Farahmand *et al*.* demonstrated that ML algorithms can perform accurate urgency triage for patients with AAP, though the study’s small sample size of 215 patients limits its significance [12]. Similarly, *Lee *et al. utilized basic information and laboratory values to predict an admission class for patients in the emergency department, achieving an AUROC of 0.97 for intensive care admission prediction [13]. A recent review by *Fernandes *et al*.* further concluded that intelligent CDSS could improve patient prioritization in emergency settings. However, limited real-world AI applications in healthcare reveal a lack of validation and implementation [14]. Notably, a gap exists between our participants’ perspectives and current evidence regarding AI’s role in CDM. On the one hand, our participants expressed moderate confidence in AI’s ability to predict postoperative mortality and adverse events (Fig. 7), which is in line with *Cobianchi *et al*.’s* insight that international emergency surgeons favor traditional CDM tools [8]. On the other hand, high-quality studies by *Bihorac *et al*.* and *Mahajan *et al*.* demonstrated that ML algorithms can predict postoperative mortality with AUROC values ranging from 0.77 to 0.83 and 0.95 to 0.97, respectively. Their algorithms also achieved AUROC values between 0.82 and 0.94 for predicting postoperative complications, including neurologic and cardiovascular events [15, 16]. For emergency surgery, *Bertsimas *et al*.’s* ML algorithm achieved an AUROC of 0.92 for mortality prediction and 0.84 for morbidity, outperforming traditional scoring systems [17]. Additionally, two recent studies indicate that ML algorithms outperform surgeons in predicting postoperative outcomes [5, 18], and the ACS NSQIP risk calculator has transitioned from a regression-based approach to a ML model to improve accuracy [19]. Based on this evidence and guidelines recommending the use of risk calculators, we encourage surgeons to familiarize themselves with these ML tools for optimized patient care [20]. Both risk calculators and organizational support applications demonstrate desirable roles for AI in CDM. While our findings represent a current snapshot, and opinions may evolve, participants’ perspectives suggest that further research should focus on increased AI implementation and stronger evidence to support its clinical utility.

### Differences in the surgical community

To better understand the causes of the abovementioned discrepancies, we focus on the characteristics of our participants. Like the findings of *Cobianchi *et al*.*, we observed a broad spectrum of opinions on AI for CDM. The authors concluded that both enthusiastic early adopters and those who favor traditional methods play a role in the surgical community [8]. Our data suggest that attitudes toward AI may vary by age and hospital type: younger male participants working in tertiary (academic) hospitals appear to view AI’s potential in CDM more favorably and express fewer concerns. This pattern aligns with studies from the USA, Germany, and the Netherlands, where younger, male, and academic individuals of the general population tend to hold a more favorable view of AI’s implementation in medicine [11, 21, 22]. Conversely, *Cobianchi *et al*.’s* results show that residents do not report higher use of AI in CDM compared to more experienced surgeons [8]. The reasons behind this and the reluctance observed among other demographic groups remain unclear and warrant further investigation. In addition to demographic factors, personal knowledge and understanding of AI may also influence these attitudes, although our survey did not address this aspect. Interestingly, previous studies have shown that both surgeons and medical students have an equally limited understanding of AI’s technical principles [8, 23], potentially due to minimal exposure to AI topics in medical curricula [24]. The survey by *Pinto Dos Santos *et al. found that over two-thirds of medical students agree on the necessity of incorporating AI training into medical education [23]. Consequently, we see a strong need for educational initiatives on AI in the surgical community, beginning in medical training. Professional societies should also develop appropriate curricula, and guidelines are needed to establish a clear framework for AI’s use in CDM [24]. Ultimately, combining structured AI training with the development of robust algorithms is essential to foster trust in this new technology. While our study may be subject to some selection bias (e.g., limited representativeness of the German surgical demographic), our findings suggest that skeptics and their concerns should be actively included in this process.

### Concerns and ethical issues

Overall, our participants largely accepted the use of AI in CDM, as only 19% indicated that AI should not be involved. However, respondents agreed on various practical and ethical concerns regarding AI’s implementation in CDM. It should be noted that we predefined the items, and participants could only indicate their level of agreement, not describe their concerns in detail. The most agreed on concern (Fig. 4), was the potential loss of physicians’ clinical judgment. This issue, that the availability of technical aids may negatively affect doctors’ clinical judgment, was documented by *Ersoydan *et al*.*, who observed a significant decline between 2005 and 2019 in the accordance between suspected diagnoses on abdominal computed tomography scan requests and final diagnoses, alongside a concurrent increase in radiological examinations [25]. Thus, the concerns highlighted by our participants appear to be valid and cannot be disregarded. Additionally, most of our participants indicated concerns about the transparency of AI, as it could become challenging to understand how AI-generated outputs are reached, and that the use of AI could decrease patient trust in surgical CDM (Fig. 4). A Deloitte survey similarly found that the German public is strongly concerned about nontransparent decision-making [11]. Furthermore, *Lennartz *et al*.* found that German patients are uncomfortable with the idea of AI making therapy decisions without review by a physician [26]. In the USA, 60% of the public are uneasy with the idea of their doctors relying on AI, and 57% believe that the doctor-patient relationship could deteriorate with AI’s use [21]. Consequently, a review by *Tucci *et al. concluded that establishing trust in medical AI applications requires a careful balance between algorithmic complexity, comprehensibility, and transparency [27]. The appropriateness of trust in the context of medical AI is currently debated. *DeCamp and Tilburt* deny this and define trust in the narrower sense as a patient placing their fate in the hands of a doctor in a vulnerable situation, thereby creating an interpersonal connection [28]. In contrast, reliance may be a more appropriate term for discussing human-AI relationships. *Kerasidou *et al. therefore propose a shift in the AI debate from trust to reliance [29]. While our study did not empirically explore these ethical dimensions, our findings suggest that reliance is a crucial factor for the effective implementation of AI in CDM. To foster this reliance, it is essential for surgical scientists to critically assess AI tools through applied research, generating validated experience and identifying potential biases and limitations. In clinical practice, the physician–patient relationship remains paramount, as it forms the basis of interpersonal trust [30]. Ultimately, we view AI tools, such as intelligent CDSS, as supplements rather than replacements for the human element in medicine.

### Possible implementation of AI in CDM for AAP

Building on the preceding discussion, we explore how AI might be effectively implemented in CDM for AAP. It should be noted that we did not fully assess practical challenges associated with the use of AI, such as integration into clinical workflows and friendly user interface designs. The application of guidelines and the latest scientific evidence remains essential for optimal emergency care. Participants in our study, however, rated CDM for AAP as highly dependent on individual experience, poorly standardized, and less evidence-based (Fig. 5). Similarly, the survey by *Cobianchi *et al. of the international surgical community identified challenges in emergency CDM, including incomplete data and a dependence on personal beliefs [8]. In contrast, German patients surveyed by *Lennartz *et al. viewed AI as superior to human doctors in applying the latest scientific evidence [26]. Based on this, we believe that the implementation of AI in CDM can address the following problems in traditional CDM: firstly, ML algorithms could ensure structured and standardized data collection by identifying missing variables and prompting physicians to gather additional findings as needed. Secondly, from a clinical perspective, AI could help surgeons to compare collected data with the latest evidence to enhance CDM. This view aligns with conclusions from *Cobianchi *et al*.*, who saw potential in AI for enabling surgeons to validate their own decision with AI’s recommendations [8]. In fact, a recent review by *Fernandes *et al*.* showed that intelligent CDSS, validated in the emergency department, improved consistency and reliability in physicians’ CDM. In contrast, specific research is yet needed to validate this assumption [14]. In addition to effective data collection and evidence-based recommendations, formulating an accurate initial diagnosis is central to the clinical management of AAP [2]. In our study, 46% of participants expressed confidence in AI’s ability to make a correct diagnosis for AAP (Fig. 7). Like all our results, this should be seen in relation to the other items and therefore indicates a rather mediocre level of confidence. Correspondingly, a double-blind, prospective study by *Faqar-Uz-Zaman *et al. showed that traditional doctor-patient interactions outperformed AI in diagnostic accuracy for AAP (81% vs. 52%, *P* < 0.001). Our findings similarly emphasize the importance of physical examination, which participants rated as the most critical component of CDM in AAP (Fig. 6). Yet, the combined accuracy of doctors and AI, at 87%, and the reduction in complications through earlier diagnosis exemplify the potential advantages of AI [31]. To us, future AI developments should prioritize human factors and interpersonal interaction, while data privacy and environmental impact were of less concern to our participants (Fig. 4). Provided that essential factors such as safety and usability are addressed, we anticipate that AI applications for AAP could enhance medical care by enabling faster, more reliable treatments.

## Conclusion

Our survey explores German surgeons’ views on AI in CDM, especially for AAP. Participants recognized AI’s organizational potential, such as for urgency triage and workload management. Scientific studies confirm AI’s accuracy in areas like urgency triage and mortality prediction, but real-world application remains limited due to validation and implementation gaps. While many participants were cautious about AI’s clinical predictive power, studies show that AI can reliably predict postoperative outcomes, outperforming traditional CDM tools. Participant's attitudes vary with demographic factors; younger and academic participants were generally more receptive to AI. This aligns with trends in the general population, and lacking technical AI knowledge could be an additional factor. We therefore advocate integrating AI education into medical training, supported by professional guidelines, to build trust and competence. Ethical concerns of our participants included loss of clinical judgment and transparency in AI decisions, which may undermine patient trust. Thus, fostering “reliance” rather than “trust” in AI could frame its role as a supportive tool, not a replacement for physician–patient relationships. For AAP management, AI could standardize data collection and enhance evidence-based care, though studies suggest traditional methods still excel in diagnostic accuracy. Future AI advancements should emphasize human-centered design, ensuring integration without compromising clinical judgment or patient relationships, ultimately enhancing CDM’s reliability and efficiency.

## Supplementary Information

Below is the link to the electronic supplementary material.

Supplementary file1 (XLSX 15 KB)

Supplementary file2 (XLSX 17 KB)
